# Recent Advances in the Development of Portable Electrochemical Sensors for Controlled Substances

**DOI:** 10.3390/s23063140

**Published:** 2023-03-15

**Authors:** Zhaohua Dai

**Affiliations:** Forensic Science Program, Department of Chemistry and Physical Sciences, Pace University, New York, NY 10038, USA; zdai@pace.edu; Tel.: +1-212-346-1760

**Keywords:** electrochemical, sensors, portable, controlled substances, screen-printed electrodes, aptamer

## Abstract

This review article summarizes recent achievements in developing portable electrochemical sensing systems for the detection and/or quantification of controlled substances with potential on-site applications at the crime scene or other venues and in wastewater-based epidemiology. Electrochemical sensors employing carbon screen-printed electrodes (SPEs), including a wearable glove-based one, and aptamer-based devices, including a miniaturized aptamer-based graphene field effect transistor platform, are some exciting examples. Quite straightforward electrochemical sensing systems and methods for controlled substances have been developed using commercially available carbon SPEs and commercially available miniaturized potentiostats. They offer simplicity, ready availability, and affordability. With further development, they might become ready for deployment in forensic field investigation, especially when fast and informed decisions are to be made. Slightly modified carbon SPEs or SPE-like devices might be able to offer higher specificity and sensitivity while they can still be used on commercially available miniaturized potentiostats or lab-fabricated portable or even wearable devices. Affinity-based portable devices employing aptamers, antibodies, and molecularly imprinted polymers have been developed for more specific and sensitive detection and quantification. With further development of both hardware and software, the future of electrochemical sensors for controlled substances is bright.

## 1. Introduction

The illegal use of controlled substances has various detrimental impacts on individuals, families, and the whole of society. Relevant people and authorities are rightfully concerned. Quick, affordable, and reliable tools and methods are needed to detect such substances under certain circumstances so that early intervention can be deployed, and relevant laws can be justifiably and timely enforced while preventing or minimizing injustice and unnecessary stigmatization. Over the years, many methods have been proposed and/or developed [1,2,3,4,5,6,7,8,9,10,11]. Although color tests for drugs are quick, and they are still being used by law enforcement agencies and sometimes even by the general public [12,13,14], false results from such presumptive tests are not that uncommon. What is more, color test reagents are often corrosive, toxic, and/or carcinogenic, which makes them unfriendly to the users and the environment. Although the workhorse and gold standard of forensic drug analysis, gas chromatography in tandem with mass spectrometry (GC-MS) [15,16,17,18], produces reliable results, highly trained personnel are needed to operate the often bulky and expensive instruments that are connected with (helium) gas cylinders and vacuum pumps, making them not suitable for field operations, especially high-throughput field operations. High-performance liquid chromatography coupled with (tandem) mass spectrometers (LC-MS or LC-MS/MS) can be quite useful [19,20,21,22,23], although they are not portable either. Hand-held spectrometers, including portable Raman spectrometers, have been used for field operations [24,25,26]. However, they face a number of challenges. A number of issues are in the process of being tackled by specialized Raman methods [27,28,29], some of which are summarized in a recent review by this author [30]. Another promising and seemingly more straightforward, cost-effective, and accurate way to solve such problems is using electrochemical sensors [31,32,33]. A lot of effort has been put into developing electrochemical sensors for a variety of drugs, and they are included in several reviews [11,34,35,36,37,38,39,40,41,42,43,44]. This review article focuses on recent developments within the last few years of portable electrochemical devices for the detection and/or quantification of controlled substances with potential on-site applications at the crime scene or other venues.

## 2. Sensing Using Commercially Available Carbon Screen-Printed Electrodes and Portable Potentiostats

Carbon screen-printed electrodes (SPEs) are very suitable for the electrochemical analysis of many analytes using portable devices [45]. They are commercially available or can be fabricated easily in-house at very affordable costs. Many illegal drugs contain groups, such as various amino groups, that are electroactive in the potential window of simple carbon SPEs, producing peaks in square wave voltammetry (SWV) and/or differential potential voltammetry (DPV) scans. Indeed, carbon SPEs or their close analogs have been used in portable electrochemical sensing systems for controlled substances.

**Figure 1:**
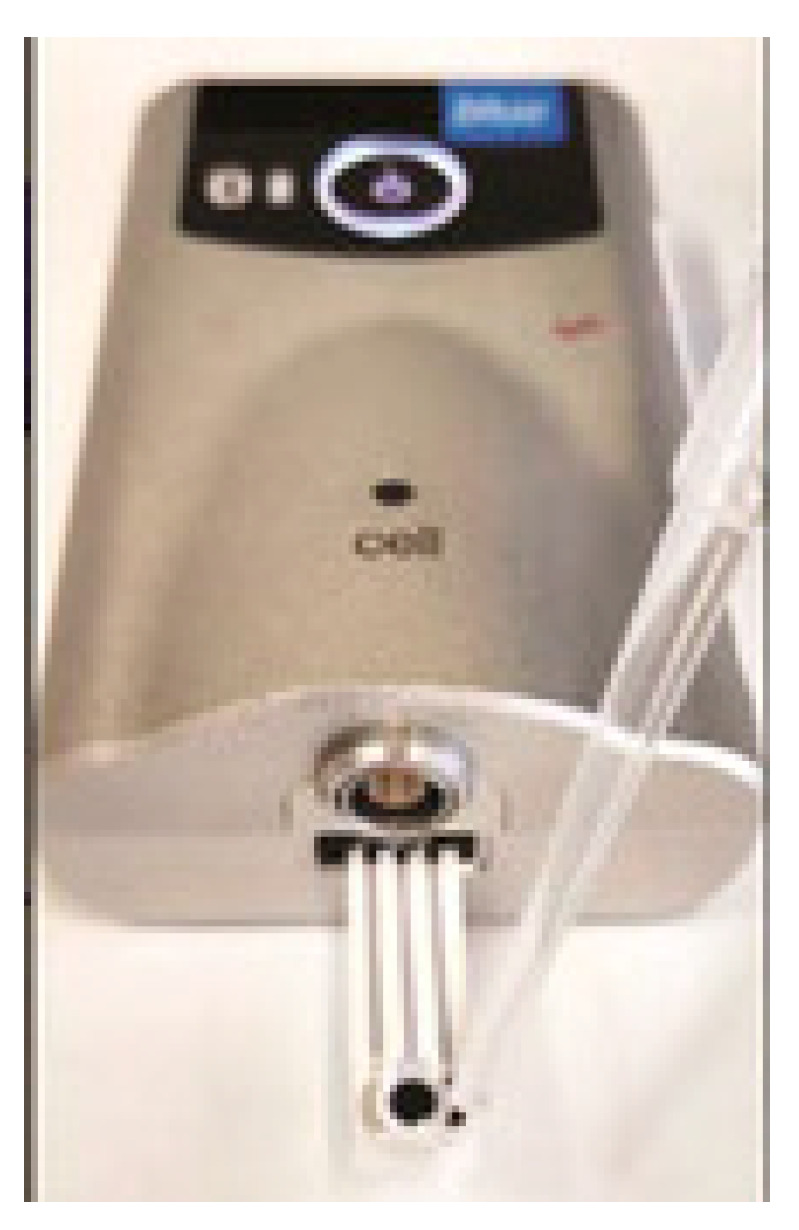
A portable electrochemical device for on-site detection of illicit drugs. The deposition of the solution on its connected SPE is shown [46]. Adapted from MDPI under a Creative Common CC BY license.

A Belgian research group utilized commercially available portable potentiostats and carbon SPEs to construct electrochemical devices and developed some methods for the quick and accurate detection of illegal controlled substances [46,47]. These devices and methods seem to be quite cost-effective, and the authors developed them for possible uses at important entry points, such as border crossings and customs, and venues prone to illicit drug use, such as music festivals. The SPEs were disposable. Each SPE used was a three-electrode system made of ordinary materials without further decoration. The working electrode material was graphite (of course, carbon in nature) with a diameter of 3 mm, and the counter electrode material was carbon. There was also a (pseudo) silver reference electrode in each SPE. Some SPEs were pre-anodized for certain measurements and targets. To facilitate on-site or in-field investigations, kits containing a potentiostat (MultiPalmSens4 or EmStat Pico with PSTrace/MultiTrace software from PalmSens, Houten, The Netherlands) with Bluetooth connectivity, SPEs, small vials of buffers, disposable plastic spatulas, and pipettes could be prepared for ready use. The SPE was inserted into the potentiostat’s SPE connector, as shown in Figure 1. The potentiostat was then connected with a user-friendly interface via Bluetooth. Subsequently, a small amount of powdery evidence was transferred into a vial of a buffer using a disposable spatula. After vigorous shaking, a drop of the solution was deposited directly on the round working electrode of the SPE using a disposable pipette, as shown in Figure 1. 

Launching a predeveloped SWV method from the interface, the operation started, and data were collected and analyzed to produce results. The whole process sounds straightforward enough, and law enforcement officers should be able to use them on-site or in-field without the need for extensive training.

**Figure 2:**
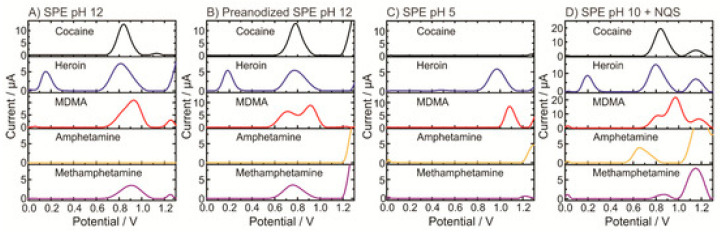
Electrochemical profiles of four illicit drugs acquired by SWV using unmodified carbon SPEs at different pHs and conditions [46]. Reused from MDPI under a Creative Common CC BY license.

For potential application at borders and customs [46], the electrochemical profiles (EPs) of pure cocaine, heroin, MDMA, and amphetamine (Figure 2), as well as some common cutting agents under four different conditions (pH 12, pre-anodized SPE in pH 12, pH 5, and pH 10 with 1, 2-naphthoquinone-4-sulfonate (NQS)) were acquired using square wave voltammetry (SWV) to build an EP library and a tailor-made script for drug identification. Clearly, the electrochemical profiles of different compounds were different and dependent on measuring conditions, especially the pH. Amphetamine must be derivatized, in this case with NQS, through simple mixing and measured in the pH 10 buffer to obtain a peak in the potential window of the cost-effective disposable carbon SPEs [48]. Although the EP library was constructed using four different conditions on every drug and cutting agent investigated in the study, it does not mean that every unknown sample needs to be investigated using all four different conditions. In the cases where there might be some overlap, the proper identification of an unknown sample could still be achieved by probing it in two different buffers. The device was then put to real tests by examining 40 seized samples that were previously analyzed by the gold standard GC-MS at a governmental forensic institute. Each sample contained only one of the four targeted controlled substances, with or without cutting agents. The electrochemical results agreed 100% with the GC-MS results for the controlled substances in them, much better than the 50% correctness obtained using a portable Raman spectrometer, although some cutting agents were not picked up by the electrochemical method in some samples.

**Figure 3:**
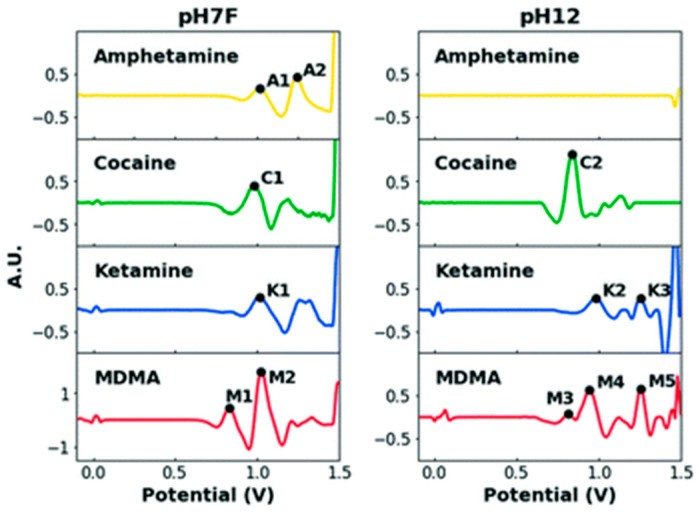
Pre-processed SWV electrochemical profiles of the four selected illicit drugs in pH 7F (a pH 7 PBS buffer containing some formaldehyde, **left**) and pH12 (**right**), with relevant peaks labeled [47]. Reused from the Royal Society of Chemistry under a Creative Commons Attribution-NonCommercial 3.0 Unported Licence.

**Figure 4:**
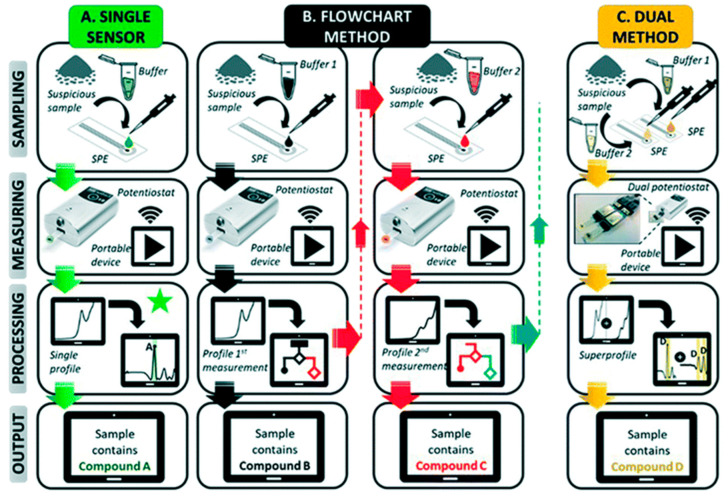
Schematic overview of three electrochemical methods for drug sensing [47]. Reused and adapted from the Royal Society of Chemistry under a Creative Commons Attribution-NonCommercial 3.0 Unported Licence.

Later on, the same PI’s research group further developed this electrochemical approach and came up with two methods for potential use at music festivals for on-site, simultaneous, rapid, and reliable detection of four popular drugs currently used illegally at such festivals in Europe: cocaine, MDMA, amphetamine, and ketamine [47]. Although they sound similar to the method developed for use at borders, they offered faster results because they utilized only two buffers instead of four, and they followed clear strategies. The two buffers used were a pH 12 PBS (0.020 M K_2_HPO4 and 0.1 M KCl), dubbed pH 12, and a pH 7 PBS (0.1 M KH_2_PO_4_, 0.1 M KCl, and 11.1% v/v formaldehyde), dubbed pH7F. The formaldehyde in pH 7F derivatized amphetamine so that it gave SWV peaks in the 0–1.5 V potential window and helped produce additional characteristic peaks for other drugs (Figure 3), which is consistent with the finding by the same PI’s group that paraformaldehyde-coated electrochemical sensors could improve amphetamine detection [33]. Since there were severe overlaps among some drugs, and amphetamine had no peaks measured in the pH 12 buffer, it was impossible to target multiple drugs with just one SPE (Figure 4A). To overcome such problems, multiple SPEs were used to develop two different methods for the fast identification of the drugs in question: a flow chart method in which two SPEs were employed sequentially to probe the evidence in two different conditions and buffers (Figure 4B) and a dual-sensor method in which two SPEs were used simultaneously in one portable electrochemical device (a MultiPalmSens4 potentiostat with PSTrace/MultiTrace software from PalmSens, Houten, The Netherlands) to measure the same evidence in two different buffers, pH 12 and pH 7F (Figure 4C). A training set of samples containing the pure drugs, commonly encountered pure cutting agents for such drugs, and binary mixtures (each containing one drug and one cutting agent), respectively, were selected, measured, and analyzed to optimize the parameters, databases, scripts, and overall data processing for both methods.

In the flowchart method, the results from the first measurement were utilized by the software to propose the conditions for a second measurement, if necessary (Figure 4B). The results of the two measurements were then linked to offer higher accuracy than the single-sensor method. Apparently, in rare cases when no second measurement is necessary, the flowchart method becomes the single-sensor method. In the dual-sensor method, the two simultaneously acquired EPs were combined into a “superprofile” (Figure 4C), generating more than double the information yielded by either individual EP (“1 + 1 > 2”) and giving much more accurate results. The two methods were applied to analyze 40 seized samples. The flowchart method yielded 80% correctness, and the dual-sensor method gave 87.5% correctness in identifying the controlled substances in them, while a portable Raman spectrometer offered only 60% correctness.

Although the two different conditions and buffers were enough for both of these two methods when there was only one of the four drugs in each sample, and regardless of the presence or absence of cutting agents, additional conditions might be needed for other drugs or more complicated samples containing multiple drugs. In the flow chart method, the information gathered from the first measurement would determine the conditions to be used in the subsequent measurement using a second SPE. Even more measurements under additional conditions using additional SPEs proposed by the software might become necessary. As for the dual-sensor method, other drugs might require other sets of conditions, which need to be optimized in advance. It might have to involve some educated guesses in choosing the combination of two conditions and buffers when analyzing “more unknown” or more complicated samples. Sometimes, three or even more SPEs and measuring conditions might have to be used in parallel.

The same P.I.’s group found out earlier that with the help of a solution mediated by the surfactant sodium dodecyl sulfate (SDS), the enhanced detection of five illicit drugs, cocaine, heroin, 3,4-methylenedioxymethamphetamine, 4-chloro-a-pyrrolidinovalerophenone, and ketamine in oral fluid, was achieved using square wave adsorption stripping voltammetry on carbon SPEs [49]. This electrochemical sensing method was very sensitive, with submicromolar LODs and wide linear ranges (~1–30 mM). It was very cost-effective since no modification or functionalization of the carbon SPEs was needed, and the surfactant, SDS, is very cheap and readily available. There was a more recent effort from the P.I.’s group to investigate the electrochemical detection of methamphetamine using square wave voltammetry on disposable graphite SPEs, resulting in very sensitive detection [50].

The portable devices (with one or more potentiostats within) were sourced from PalmSens (Houten, The Netherlands). Their sizes are conveniently small enough that other researchers have also used them or adapted them for constructing portable electrochemical devices for the sensing and analysis of illicit drugs [51,52]. They can be interfaced with computers and mobile phones or tablets easily, enabling facile data processing and analysis.

## 3. Sensing Using Lab-Fabricated or Modified SPE/SPE-like Electrodes and Portable Potentiostats

**Figure 5:**
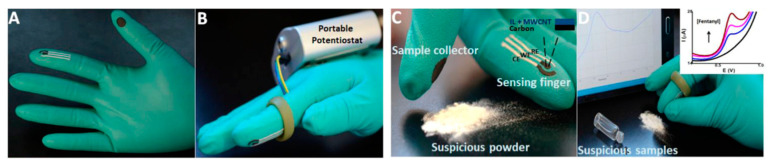
Overview of the lab-on-a-glove device for on-site electrochemical detection of fentanyl; (**A**) glove containing SPE on the sensing index finger and sample collector pad on the thumb. (**B**) The glove-based sensor with a portable electroanalyzer–potentiostat. (**C**) Collecting a powdery sample. (**D**) Joining of collecting (thumb) and sensing (index) fingers after swiping a powder sample to start SWV scans [51]. Reused with permission from Elsevier.

Some drugs, such as fentanyl, are very toxic and dangerous, even to law enforcement personnel who unknowingly come into contact with them because of work. With the intention to equip such personnel with on-site sampling and detection tools for fentanyl, a research group developed an electrochemical sensor using carbon SPEs printed on disposable gloves and a portable potentiostat sourced from, as mentioned earlier, PalmSens, which wirelessly interfaced with a smartphone or a tablet for data acquisition, processing, and analysis (Figure 5) [51]. First, 3-D printed thumb and index finger molds were inserted into the corresponding positions in disposable nitrile gloves to facilitate smooth screen printing. On the molded index finger part of the glove was printed a layer of Ag/AgCl as the reference electrode (RE) and a layer of carbon as the working electrode (WE) and counter electrode (CE), respectively, followed by finishing procedures. On the thumb finger part of the glove, a circular carbon pad (1 cm in diameter) was similarly printed for collecting samples. A composite made of multiwall carbon nanotubes (MWCNT), 4-(3-Butyl-1-imidazolio)-1-butanesulfonate (an ionic liquid, IL), and polyethylenimine (PEI) was drop-casted on the WE and airdried to be ready for analyzing the liquid samples. For powder analysis, a conductive semi-solid hydrogel was applied to cover the surface of the SPE. For fentanyl sensing, the investigator put on the glove and attached the SPE via a ring and wires to a portable EmStat3 Blue device (PalmSens, Houten, The Netherlands) secured on the back of the hand. The sample was swiped with the printed carbon pad on the collecting (thumb) finger, which was then joined with the SPE on the sensing (index) finger before an SWV scan was initiated by a program, and the data was transmitted wirelessly for processing and analysis to yield a result. Decorating the working electrode with the ionic liquid was found to be necessary since there was no fentanyl signal without such modification. The device could reliably detect fentanyl in both liquid and in powder. Please note that the single and distinct oxidation peak for fentanyl occurred at different voltages: +0.65 V in liquid and +0.76 V in powder. The device was quite sensitive and selective since it required only a small amount of fentanyl, in the presence or absence of commonly encountered cutting agents, for identification. Quantitative analysis of liquid samples was said to be possible. However, it is not clear if any drugs, especially other opioids, can interfere with fentanyl sensing or not, which deserves further study since small amounts of fentanyl are often mixed with heroin in current illegal drug markets. It would be nice if such wearable sensors could be used to detect other drugs.

Some safety concerns still need to be addressed to overcome the hesitance in touching drug evidence directly using gloved fingers when other sampling tools are available. With further development, such “swipe, scan, sense, and alert” wearable “lab-on-a-glove” electrochemical sensors may find acceptance and bring drug analysis directly and literally “to the user’s fingertips”, making the facile and safe detection of toxic and dangerous drugs in emergency situations possible and enabling quick, on-site but informed decision making. Wearable flexible electrochemical sensors may find applications in other important fields [53], potentially making drug detection through monitoring sweat possible.

There have been other efforts to electrochemically detect fentanyl. Casting a zinc-based metal–organic framework (Zn(II)-MOF) on the surface of a carbon SPE resulted in an electrochemical sensor that was able to detect fentanyl in an aqueous solution, urine, and plasma using DPV with very high sensitivity [54]. Its LOD was 0.3 μM, and its linear range was wide, from 1 to 100 μM.

**Figure 6:**
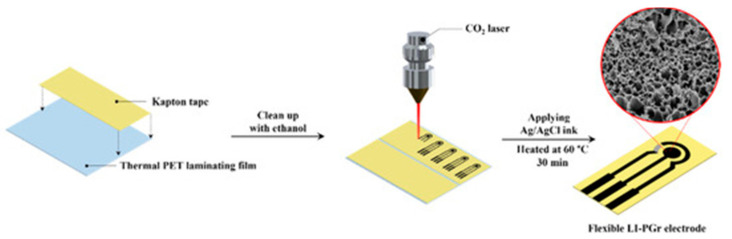
Schematic representation of the fabrication of laser-induced porous graphene electrode [52]. Reused from MDPI under a Creative Common CC BY license.

A group in Thailand found a simple and very economical way to make a portable electrochemical sensor for methamphetamine [52]. It has been known that polyimide films, even commercial ones, can be turned into porous graphene structures, which were found to be excellent electrical conductors with a very large and specific surface area [55]. As shown in Figure 6, the group attached Kapton tape made from polyimide film to polyethylene terephthalate (PET) laminating film. After cleaning, a three-electrode pattern with a WE (d= 3 mm), a CE, and an RE was directly engraved in the Kapton tape with a CO_2_ laser. The material in this engraved electrode was confirmed to be 3-D porous graphene. Afterwards, Ag/AgCl ink was painted onto the RE part to create a reference electrode to finish the construction of a highly flexible laser-induced porous graphene (LI-PGr) electrode supported by a plastic film, which looked like an SPE.

**Figure 7:**
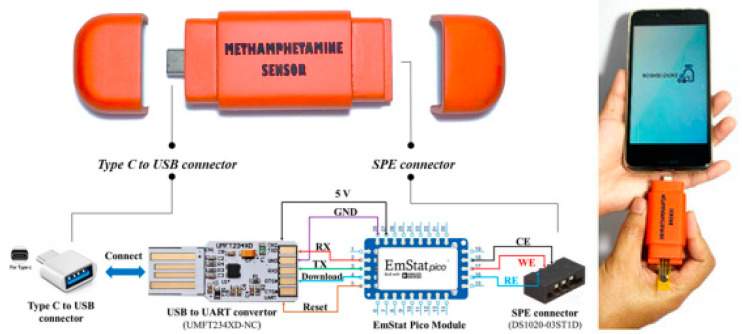
A portable electrochemical device containing a fully functional EmStat Pico potentiostat connected with a USB to a UART convertor to interface with a type-C USB connection and an SPE connector [52]. Reused from MDPI under a Creative Common CC BY license.

They attached an SPE connector and a USB connector to the two ends of a portable potentiostat (EmPico with PSTrace v 5.6 software) sourced from PalmSens (Houten, The Netherlands), respectively, and encased the setup in a 3-D printed housing, resulting in an electrochemical device that looked like a USB drive (Figure 7). The LI-PGr electrode was inserted into the SPE connector of the device. Then, the device was connected through the USB connector to a smartphone which drove the differential pulse voltammetry (DPV) analysis of methamphetamine through an app after a liquid sample was applied to the electrode.

The maximum anodic peak for methamphetamine was at + 0.4 V. The maximum anodic peak current could be used for the quantitative analysis of methamphetamine with two linear ranges: from 1.00 to 30.0 μg/mL and from 30.0 to 100 μg/mL. The sensor was highly sensitive to methamphetamine with a limit of detection (LOD) of 0.31 μg/mL; common cutting agents and inorganic ions had little to no interference with it. Such compounds and drugs as pseudoephedrine, alprazolam, and diazepam posed no interference at all. Although clonazepam produced a peak, it was clearly separated from that of methamphetamine, bringing no significant interference in a binary mixture.

As was noted earlier, the group not only made a new type of LI-PGr electrode but also constructed their own electrochemical device using an EmStat Pico potentiostat sourced from PalmSens (Houten, The Netherlands). The constructed device was linked to a cellphone through a USB connection and ran by the PSTrace program supplied by PalmSens. Since such Emstat Pico potentiostats and devices sourced from PalmSens, wirelessly connected to cellphones and run by PSTrace, were used in a study mentioned earlier [46], one might want to use such commercially available devices for sensitive and facile on-site methamphetamine detection while employing LI-PGr electrodes, either made in-house or externally sourced when they become commercially available, in order to achieve more consistent results, a wider adoption, and even further reduced costs.

## 4. Affinity-Based Electrochemical Sensors

To increase the sensitivity and specificity, electrochemical sensors can be decorated with moieties that are selective for drugs of interest. Quite some work has been conducted, and interesting results have been obtained in this direction.

**Figure 8:**
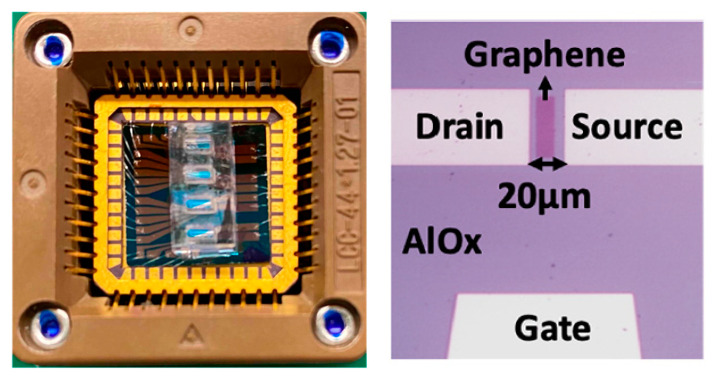
(**Left**) A G-FET chip (1.2 cm × 1.2 cm) with 4 PDMS wells. (**Right**) Microscopic image of a single G-FET with source–drain and side gate electrode and graphene-sensing window with AlOx passivation [61]. Reused and adapted with permission from the American Chemical Society.

**Figure 9:**
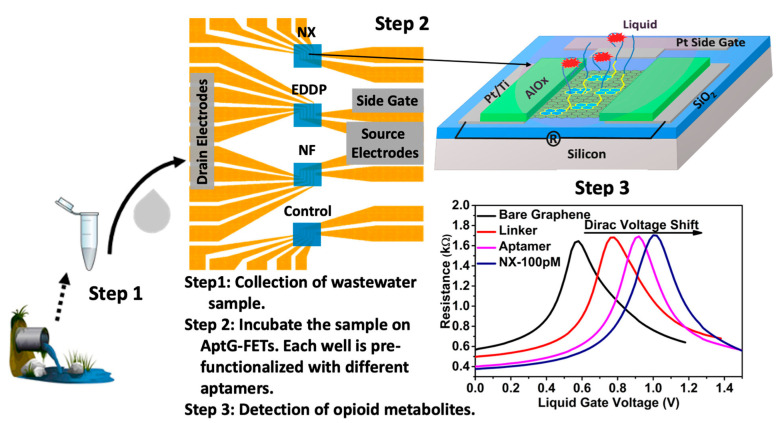
Schematic illustration of an on-site chip-based rapid detection platform for near real-time monitoring of opioid metabolites in wastewater using AptG-FET sensor technology [61]. Reused and adapted with permission from the American Chemical Society.

Wastewater-based epidemiology (WBE) can help gather important intelligence on neighborhood-wide illegal consumption of controlled substances and help create appropriate remedial policies and tools in terms of fighting crimes and improving public health [56,57,58,59,60]. For such purposes, sensitive, cheap, and easy-to-use tools for monitoring relevant drugs and their metabolites in wastewater are needed. Recently, a miniaturized aptamer-based graphene field effect transistor (AptG-FET) platform capable of the simultaneous on-site detection of several opioid metabolites in wastewater was reproducibly constructed through a facile and cost-effective process [61]. Included in the 5 cm × 8 cm platform was a 1.2 cm × 1.2 cm single chip fabricated in the researchers’ lab, which consisted of four different sets of five G-FET (4 × 5) devices. A 1.5 × 1.2 mm PDMS well was mounted on each of the four sets, segregating them (Figure 8). The PDMS wells also served the function of holding solutions for functionalizing the graphene-sensing parts of the devices and for measurements. Aptamers highly specific and selective for three different opioid metabolites, noroxycodone (NX), 2-ethylidene-1,5-dimethyl-3,3-diphenyl- pyrrolidine (EDDP), and norfentanyl (NF), were carefully selected and validated to functionalize three of the four sets of devices, respectively, on the chip. The fourth set was reserved for the control and, therefore, was not functionalized. The electrical resistance of the graphene was highly sensitive to its surface environment, as shown in the resistance vs. liquid gate voltage plots. After each effective decoration, there was a Dirac voltage shift. The Dirac voltage of each aptamer-decorated device could be established experimentally first. Then, the wastewater samples were added to each PDMS well (Figure 9). After the effective binding of an aptamer with its targeted opioid metabolite, there was a further Dirac voltage shift. Therefore, the identity of the opioid metabolite could be established depending on which set of devices produced such a Dirac voltage shift. If and when the Dirac shifts were produced in multiple sets of devices, the identities of the metabolites could be simultaneously found out accordingly. What is more, the magnitude of the Dirac voltage shifts depended on the concentration of the targeted analyte. Therefore, a quantitative analysis could be achieved. Since the signals from the five G-FET devices in each PDMS well were obtained simultaneously and averaged for data analysis, the results were fast and more reliable. The devices were highly sensitive, showing LODs of 38, 27, and 42 pg/mL for NX, EDDP, and NF, respectively, very suitable for the analysis of these metabolites in wastewater whose concentrations are in the range of pg/mL to ng/mL. The whole measurement setup, composed of the entire platform, a digital multimeter for measuring the resistance (0−5 kΩ), and a 0−2 V voltage supply, could be easily miniaturized and made portable for in-field analysis. Of course, there are potentially many more drugs–metabolites to monitor in wastewater, which was expected to be easily achieved by employing a bigger wafer where many more chips (for example, 100 chips on a 6-in wafer) could be constructed, and the whole setup would still be portable. Additionally, for WBE and other applications, devices that can monitor drugs and their metabolites continuously [62] and reagentlessly are highly desirable. It is unclear if such aptamer-based devices can do that. If the affinities of the aptamers to the drugs–metabolites are too high, they might not be able to continuously follow the concentration fluctuation of the drugs–metabolites in the wastewater flow in real-time.

**Figure 10:**
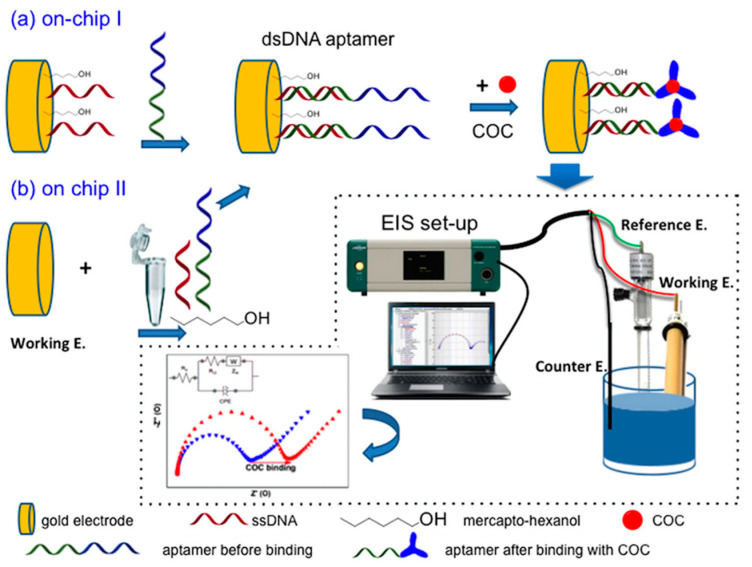
Schematic illustration of the DDIAS for detection of cocaine within the ‘on-chip I’ (**a**) and ‘on-chip II’ (**b**) approaches using EIS [63]. Reused from Springer Nature under the Creative Commons CC BY license.

In an earlier WBE attempt, an aptamer was immobilized onto a gold electrode through a DNA-directed process for sensing cocaine in sewage [63]. Two different “on-chip” approaches to immobilizing the aptamer, both using the same materials but in different procedures, were explored (Figure 10). The detection was achieved through electrochemical measurements using electrochemical impedance spectroscopy (EIS) with the ferri–ferrocyanide couple, [Fe(CN)_6_]^3−/4−^, as the redox marker, which needed to overcome an electrostatic barrier in order to exchange an electron with the electrode. In the absence of cocaine, the DNA aptamer assumed a two-stem loop conformation. In the presence of cocaine, the aptamer bound it to form a three-way junction, causing an increase in the electrostatic barrier, which was manifested in the measured charge transfer resistance (*R*ct) of the system. The “on-chip approach II”, in which a thiolated single-stranded DNA (ssDNA) probe was initially hybridized with an aptamer tailed with a complementary ssDNA in a solution to form an aptamer tailed with a double-stranded DNA (dsDNA), followed by co-immobilization with 6-mercapto-hexanol onto the gold electrodes, worked much better. This DNA-directed immobilization aptamer sensor (DDIAS) was optimized, and it was found to be very specific, sensitive, and quantitative toward cocaine with an LOD of 10 nM.

An affinity-based electrochemical sensor for THC in saliva using EIS was constructed on PET substrates by depositing electrode patterns with 125 nm of Au on it, employing the e-beam deposition technique and decorating the gold electrodes with a capture antibody for the THC-BSA hapten [64]. After non-faradaic EIS data were collected, a binary classification system, developed and implemented using appropriate algorithms, was able to predict the presence of THC in human saliva using the data. Integrating the electrochemical sensor with low-power electronics and a portable saliva swab could result in a hand-held platform for the rapid (within 1 min) detection of THC in saliva, especially in suspected driving under the influence (DUI) cases, with high accuracy. Quantitative analyses could be possible through regression analysis. The researchers strongly believed that the setup could potentially be miniaturized and become portable.

Cheap and disposable three-electrode ceramic-based chip sensors for some drugs were fabricated by grafting their carbon paste working electrode with molecularly imprinted polymers (MIPs) templated with the drugs of interest [65]. Although they were intended for therapeutic drug monitoring, one of the drugs studied, phenobarbital, is a controlled substance, and the concept is applicable to other controlled substances. Drug sensing was investigated using differential pulse voltammetry (DPV) in which the response currents were linear with the drug concentrations. Each measurement was completed in less than 2 minu. MIPs templated with a drug imparted specificity and selectivity for that specific drug, minimizing interferences. The MIPs also improved such sensors’ sensitivity.

## 5. Conclusions

Electrochemical sensors have been shown to be able to quickly and reliably detect and quantify illicit controlled substances. Miniaturization, high affordability, high reliability, fast responses, and high throughput are their future and the keys to their application in the fields of forensic science and individual and public health. Portable electrochemical devices employing cost-effective disposable carbon screen-printed electrodes may be very useful for in-field and onsite high-throughput analysis, enabling law enforcement personnel or agencies to make fast and informed decisions out of necessity under a variety of circumstances. Although devices employing unmodified or minimally modified carbon SPEs are desirable for a number of reasons and are effective for a single drug or several drugs, their abilities to differentiate a wide variety and a large number of drugs and cutting agents still need to be investigated and improved, considering drug abuse is now an epidemic and a public health crisis, at least in quite a number of jurisdictions. Further hardware development is needed to make them produce sharper and more distinct signals to generate higher resolving powers so that they can be used to detect and differentiate many more controlled substances with minimal false negatives and false positives. Further software improvement, including the employment of artificial intelligence, is likely needed to help achieve such goals. Affinity-based electrochemical sensors, employing aptamers, antibodies, and molecularly imprinted polymers, can be more specific and sensitive, although they are more complicated and quite possibly more expensive than simple carbon SPE-based sensors, especially when arrays of such sensors are needed for the detection, differentiation, and quantification of more and more controlled substances. It is expected that there will be further studies and development of such sensors to make them more suitable for the continuous monitoring of drugs and their metabolites in wastewater, offering neighborhood- and community-wide illicit drug use information and intelligence in real-time. Such wastewater-based epidemiology (WBE) investigations help create appropriate remedial policies and tools in terms of fighting crimes and improving public health. Although there are still some issues to be addressed, it is not a stretch to say that electrochemical sensors for illicit drugs will find wide application and acceptance in the forensic science field and other related fields in the near future, considering the exciting pace of innovation and development in this direction.

## Data Availability

Not applicable.

## References

[1] Liu L., Grillo F., Canfarotta F., Whitcombe M., Morgan S.P., Piletsky S., Correia R., He C., Norris A., Korposh S. (2021). Carboxyl-fentanyl detection using optical fibre grating-based sensors functionalised with molecularly imprinted nanoparticles. Biosens. Bioelectron..

[2] Truta F., Florea A., Cernat A., Tertis M., Hosu O., de Wael K., Cristea C. (2020). Tackling the Problem of Sensing Commonly Abused Drugs Through Nanomaterials and (Bio)Recognition Approaches. Front. Chem..

[3] Burks R.M., Pacquette S.E., Guericke M.A., Wilson M.V., Symonsbergen D.J., Lucas K.A., Holmes A.E. (2010). DETECHIP^®^: A Sensor for Drugs of Abuse*. J. Forensic Sci..

[4] Smith A., Wilson M.V., Trauernicht M., Holmes A.E., Jackson A. (2012). Improved image analysis of DETECHIP(^®^) allows for increased specificity in drug discrimination. J. Forensics Res..

[5] Kangas M. (2017). A New Possible Alternative Colorimetric Drug Detection Test for Fentanyl. Org. Med. Chem. Int. J..

[6] Mao K., Zhang H., Pan Y., Zhang K., Cao H., Li X., Yang Z. (2020). Nanomaterial-based aptamer sensors for analysis of illicit drugs and evaluation of drugs consumption for wastewater-based epidemiology. TrAC Trends Anal. Chem..

[7] Yoho J.N., Geier B., Grigsby C.C., Hagen J.A., Chávez J.L., Kelley-Loughnane N. (2017). Cross-Reactive Plasmonic Aptasensors for Controlled Substance Identification. Sensors.

[8] Minami T., Esipenko N.A., Akdeniz A., Zhang B., Isaacs L., Anzenbacher P. (2013). Multianalyte Sensing of Addictive Over-the-Counter (OTC) Drugs. J. Am. Chem. Soc..

[9] Penido C.A., Pacheco M.T., Zângaro R.A., Silveira L. (2015). Identification of different forms of cocaine and substances used in adulteration using near-infrared Raman spectroscopy and infrared absorption spectroscopy. J. Forensic Sci..

[10] Jones L.E., Stewart A., Peters K.L., McNaul M., Speers S.J., Fletcher N.C., Bell S.E.J. (2016). Infrared and Raman screening of seized novel psychoactive substances: A large scale study of >200 samples. Analyst.

[11] Dagar M., Yadav S., Sai V.V.R., Satija J., Bhatia H. (2022). Emerging trends in point-of-care sensors for illicit drugs analysis. Talanta.

[12] Philp M., Shimmon R., Tahtouh M., Fu S. (2018). Color Spot Test As a Presumptive Tool for the Rapid Detection of Synthetic Cathinones. J. Vis. Exp..

[13] Elkins K.M., Weghorst A.C., Quinn A.A., Acharya S. (2017). Colour quantitation for chemical spot tests for a controlled substances presumptive test database. Drug Test Anal..

[14] Hafer K.E., Brettell T.A. (2006). Presumptive Color Tests of Seized Drugs. Encyclopedia of Analytical Chemistry.

[15] Lehrer M. (1998). The role of gas chromatography/mass spectrometry. Instrumental techniques in forensic urine drug testing. Clin. Lab. Med..

[16] Orfanidis A., Krokos A., Mastrogianni O., Gika H., Raikos N., Theodoridis G. (2022). Development and Validation of a Single Step GC/MS Method for the Determination of 41 Drugs and Drugs of Abuse in Postmortem Blood. Forensic Sci..

[17] Ferreira A.B., Lobo Castro A., Tarelho S., Domingues P., Franco J.M. (2023). GC-MS—Still standing for clinical and forensic analysis: Validation of a multidrug method to detect and quantify illicit drugs. Aust. J. Forensic Sci..

[18] Brettell T.A., Lum B.J., Musah R.A. (2018). Analysis of Drugs of Abuse by Gas Chromatography–Mass Spectrometry (GC-MS). Analysis of Drugs of Abuse.

[19] Sulej-Suchomska A.M., Klupczynska A., Dereziński P., Matysiak J., Przybyłowski P., Kokot Z.J. (2020). Urban wastewater analysis as an effective tool for monitoring illegal drugs, including new psychoactive substances, in the Eastern European region. Sci. Rep..

[20] Gallardo E., Barroso M., Queiroz J.A. (2009). LC-MS: A powerful tool in workplace drug testing. Drug Test. Anal..

[21] Yagihashi G., Tarui T., Miyagi H., Ohnishi H., Watanabe T., Yamaguchi Y. (2020). Diagnostic accuracy for drug detection using liquid chromatography/mass spectroscopy in overdose patients. Acute Med. Surg..

[22] Lee J., Park J., Go A., Moon H., Kim S., Jung S., Jeong W., Chung H. (2018). Urine Multi-drug Screening with GC-MS or LC–MS-MS Using SALLE-hybrid PPT/SPE. J. Anal. Toxicol..

[23] Merone G.M., Tartaglia A., Rossi S., Santavenere F., Bassotti E., D’Ovidio C., Bonelli M., Rosato E., de Grazia U., Locatelli M. (2021). Fast Quantitative LC-MS/MS Determination of Illicit Substances in Solid and Liquid Unknown Seized Samples. Anal. Chem..

[24] Harper L., Powell J., Pijl E.M. (2017). An overview of forensic drug testing methods and their suitability for harm reduction point-of-care services. Harm Reduct. J..

[25] de Oliveira Penido C.A.F., Pacheco M.T.T., Lednev I.K., Silveira L. (2016). Raman spectroscopy in forensic analysis: Identification of cocaine and other illegal drugs of abuse. J. Raman Spectrosc..

[26] Hargreaves M. (2012). Drugs of Abuse—Application of Handheld FT-IR and Raman Spectrometers. Infrared and Raman Spectroscopy in Forensic Science.

[27] Zhang B., Hou X., Zhen C., Wang A.X. (2021). Sub-Part-Per-Billion Level Sensing of Fentanyl Residues from Wastewater Using Portable Surface-Enhanced Raman Scattering Sensing. Biosensors.

[28] Mao K., Yang Z., Zhang H., Li X., Cooper J.M. (2021). Paper-based nanosensors to evaluate community-wide illicit drug use for wastewater-based epidemiology. Water Res..

[29] Razlansari M., Ulucan-Karnak F., Kahrizi M., Mirinejad S., Sargazi S., Mishra S., Rahdar A., Díez-Pascual A.M. (2022). Nanobiosensors for detection of opioids: A review of latest advancements. Eur. J. Pharm. Biopharm..

[30] Mojica E.-R., Dai Z. (2022). New Raman spectroscopic methods’ application in forensic science. Talanta Open.

[31] Kimmel D.W., LeBlanc G., Meschievitz M.E., Cliffel D.E. (2012). Electrochemical Sensors and Biosensors. Anal. Chem..

[32] Simões F.R., Xavier M.G., Da Róz A.L., Ferreira M., de Lima Leite F., Oliveira O.N. (2017). 6—Electrochemical Sensors. Nanoscience and its Applications.

[33] Schram J., Parrilla M., Slosse A., Van Durme F., Åberg J., Björk K., Bijvoets S.M., Sap S., Heerschop M.W.J., De Wael K. (2022). Paraformaldehyde-coated electrochemical sensor for improved on-site detection of amphetamine in street samples. Microchem. J..

[34] Shaw L., Dennany L. (2017). Applications of electrochemical sensors: Forensic drug analysis. Curr. Opin. Electrochem..

[35] Boumya W., Taoufik N., Achak M., Bessbousse H., Elhalil A., Barka N. (2021). Electrochemical sensors and biosensors for the determination of diclofenac in pharmaceutical, biological and water samples. Talanta Open.

[36] Sun Y. (2022). Research on Detection of Sterol Doping in Sports by Electrochemical Sensors: A Review. J. Anal. Methods Chem..

[37] Honeychurch K.C. (2019). Review of Electroanalytical-Based Approaches for the Determination of Benzodiazepines. Biosensors.

[38] Sanghavi B.J., Wolfbeis O.S., Hirsch T., Swami N.S. (2015). Nanomaterial-based electrochemical sensing of neurological drugs and neurotransmitters. Microchim. Acta.

[39] Boroujerdi R., Paul R. (2022). Graphene-Based Electrochemical Sensors for Psychoactive Drugs. Nanomaterials.

[40] Khorablou Z., Shahdost-fard F., Razmi H., Yola M.L., Karimi-Maleh H. (2021). Recent advances in developing optical and electrochemical sensors for analysis of methamphetamine: A review. Chemosphere.

[41] Amiri M., Imanzadeh H., Sefid-Sefidehkhan Y. (2020). An Overview on Electrochemical Sensors based on Nanomaterials for Determination of Drugs of Abuse. Curr. Drug Deliv..

[42] De Rycke E., Stove C., Dubruel P., De Saeger S., Beloglazova N. (2020). Recent developments in electrochemical detection of illicit drugs in diverse matrices. Biosens Bioelectron.

[43] Poltorak L., Sudhölter E., Puit M. (2019). Electrochemical cocaine (bio)sensing. From solid electrodes to soft junctions. TrAC Trends Anal. Chem..

[44] Blake R.S., Brown K., Dennany L. (2022). Electrochemical Methodologies for the Detection of Traditional and Emerging Illicit Drugs. ECS Meet. Abstr..

[45] García-Miranda Ferrari A., Rowley-Neale S.J., Banks C.E. (2021). Screen-printed electrodes: Transitioning the laboratory in-to-the field. Talanta Open.

[46] Parrilla M., Slosse A., Van Echelpoel R., Montiel N.F., Van Durme F., De Wael K. (2021). Portable Electrochemical Detection of Illicit Drugs in Smuggled Samples: Towards More Secure Borders. Chem. Proc..

[47] Van Echelpoel R., Schram J., Parrilla M., Daems D., Slosse A., Van Durme F., De Wael K. (2022). Electrochemical methods for on-site multidrug detection at festivals. Sens. Diagn..

[48] Parrilla M., Felipe Montiel N., Durme F., De Wael K. (2021). Derivatization of amphetamine to allow its electrochemical detection in illicit drug seizures. Sens. Actuators B Chem..

[49] Parrilla M., Joosten F., De Wael K. (2021). Enhanced electrochemical detection of illicit drugs in oral fluid by the use of surfactant-mediated solution. Sens. Actuators B Chem..

[50] Dragan A.-M., Parrilla M., Sleegers N., Slosse A., Van Durme F., van Nuijs A., Oprean R., Cristea C., De Wael K. (2023). Investigating the electrochemical profile of methamphetamine to enable fast on-site detection in forensic analysis. Talanta.

[51] Barfidokht A., Mishra R.K., Seenivasan R., Liu S., Hubble L.J., Wang J., Hall D.A. (2019). Wearable electrochemical glove-based sensor for rapid and on-site detection of fentanyl. Sens. Actuators B Chem..

[52] Saisahas K., Soleh A., Somsiri S., Senglan P., Promsuwan K., Saichanapan J., Kanatharana P., Thavarungkul P., Lee K., Chang K.H. (2022). Electrochemical Sensor for Methamphetamine Detection Using Laser-Induced Porous Graphene Electrode. Nanomaterials.

[53] Gao F., Liu C., Zhang L., Liu T., Wang Z., Song Z., Cai H., Fang Z., Chen J., Wang J. (2023). Wearable and flexible electrochemical sensors for sweat analysis: A review. Microsyst. Nanoeng..

[54] Naghian E., Marzi Khosrowshahi E., Sohouli E., Ahmadi F., Rahimi-Nasrabadi M., Safarifard V. (2020). A new electrochemical sensor for the detection of fentanyl lethal drug by a screen-printed carbon electrode modified with the open-ended channels of Zn(ii)-MOF. New J. Chem..

[55] Lin J., Peng Z., Liu Y., Ruiz-Zepeda F., Ye R., Samuel E.L.G., Yacaman M.J., Yakobson B.I., Tour J.M. (2014). Laser-induced porous graphene films from commercial polymers. Nat. Commun..

[56] Erickson T.B., Endo N., Duvallet C., Ghaeli N., Hess K., Alm E.J., Matus M., Chai P.R. (2021). “Waste Not, Want Not”—Leveraging Sewer Systems and Wastewater-Based Epidemiology for Drug Use Trends and Pharmaceutical Monitoring. J. Med. Toxicol..

[57] Hahn R.Z., do Nascimento C.A., Linden R. (2021). Evaluation of Illicit Drug Consumption by Wastewater Analysis Using Polar Organic Chemical Integrative Sampler as a Monitoring Tool. Front. Chem..

[58] Huizer M., ter Laak T.L., de Voogt P., van Wezel A.P. (2021). Wastewater-based epidemiology for illicit drugs: A critical review on global data. Water Res..

[59] Bannwarth A., Morelato M., Benaglia L., Been F., Esseiva P., Delemont O., Roux C. (2019). The use of wastewater analysis in forensic intelligence: Drug consumption comparison between Sydney and different European cities. Forensic Sci. Res..

[60] Castiglioni S., Thomas K.V., Kasprzyk-Hordern B., Vandam L., Griffiths P. (2014). Testing wastewater to detect illicit drugs: State of the art, potential and research needs. Sci. Total Environ..

[61] Kumar N., Rana M., Geiwitz M., Khan N.I., Catalano M., Ortiz-Marquez J.C., Kitadai H., Weber A., Dweik B., Ling X. (2022). Rapid, Multianalyte Detection of Opioid Metabolites in Wastewater. ACS Nano.

[62] Bian S., Zhu B., Rong G., Sawan M. (2021). Towards wearable and implantable continuous drug monitoring: A review. J. Pharm. Anal..

[63] Yang Z., Castrignanò E., Estrela P., Frost C.G., Kasprzyk-Hordern B. (2016). Community Sewage Sensors towards Evaluation of Drug Use Trends: Detection of Cocaine in Wastewater with DNA-Directed Immobilization Aptamer Sensors. Sci. Rep..

[64] Stevenson H., Bacon A., Joseph K.M., Gwandaru W.R.W., Bhide A., Sankhala D., Dhamu V.N., Prasad S. (2019). A Rapid Response Electrochemical Biosensor for Detecting Thc In Saliva. Sci. Rep..

[65] Aaryashree, Takeda Y., Kanai M., Hatano A., Yoshimi Y., Kida M. (2020). A “Single-Use” Ceramic-Based Electrochemical Sensor Chip Using Molecularly Imprinted Carbon Paste Electrode. Sensors.

